# Differential Selection on Caste-Associated Genes in a Subterranean Termite

**DOI:** 10.3390/insects13030224

**Published:** 2022-02-24

**Authors:** Julianne M. Radford, David Chen, Anna M. Chernyshova, Cambrie Taylor, Alex W. Guoth, Tian Wu, Kathleen A. Hill, Graham J. Thompson

**Affiliations:** Department of Biology, Western University, London, ON N6A 5B7, Canada.; jradfor8@uwo.ca (J.M.R.); dchen362@uwo.ca (D.C.); achernys@uwo.ca (A.M.C.); ctayl65@uwo.ca (C.T.); aguoth@uwo.ca (A.W.G.); twu075@gmail.com (T.W.); khill22@uwo.ca (K.A.H.)

**Keywords:** social insect invasions, caste differentiation, kin selection, urban entomology

## Abstract

**Simple Summary:**

Social insects can sometimes boldly invade new habitats, including areas of human habitation where they can become unwanted domestic or agricultural pests. In this study, we use molecular sequence analysis to study genetic patterns associated with the invasion and division of labour in the Eastern subterranean termite *Reticulitermes flavipes*. By studying how genes vary by caste and by population, we show that even termites invasive to a metropolitan city can still harbour plenty of genetic variation, as much or more as native termite populations. We suggest therefore that invasive termites do not necessarily suffer long term loss-of-variation upon invasion. Second, we show that genes associated in their expression with the soldier caste evolve approximately twice as fast as genes expressed by other castes of this species, regardless of from what population the castes were sampled. Why termite soldier genes evolve quickly is not known, but it seems unrelated to invasion or the invaded habitat. Given that soldiers are sterile and thus have no direct fitness, the evidence for gene-level selection in the soldier caste is an intriguing example of kin selection.

**Abstract:**

Analyzing the information-rich content of RNA can help uncover genetic events associated with social insect castes or other social polymorphisms. Here, we exploit a series of cDNA libraries previously derived from whole-body tissue of different castes as well as from three behaviourally distinct populations of the Eastern subterranean termite *Reticulitermes flavipes*. We found that the number (~0.5 M) of single nucleotide variants (SNVs) was roughly equal between nymph, worker and soldier caste libraries, but *d_N_/d_S_* (ratio of nonsynonymous to synonymous substitutions) analysis suggested that some of these variants confer a caste-specific advantage. Specifically, the *d_N_/d_S_* ratio was high (~4.3) for genes expressed in the defensively specialized soldier caste, relative to genes expressed by other castes (~1.7–1.8) and regardless of the North American population (Toronto, Raleigh, Boston) from which the castes were sampled. The populations, meanwhile, did show a large difference in SNV count but not in the manner expected from known demographic and behavioural differences; the highly invasive unicolonial population from Toronto was not the least diverse and did not show any other unique substitution patterns, suggesting any past bottleneck associated with invasion or with current unicoloniality has become obscured at the RNA level. Our study raises two important hypotheses relevant to termite sociobiology. First, the positive selection (*d_N_/d_S_* > 1) inferred for soldier-biased genes is presumably indirect and of the type mediated through kin selection, and second, the behavioural changes that accompany some social insect urban invasions (i.e., ‘unicoloniality’) may be detached from the loss-of-diversity expected from invasion bottlenecks.

## 1. Introduction

The study of social insect biology often uses behavioural genetics theory to better understand the evolution of castes and associated social behavior. Inclusive fitness theory provides a resident body of work that explains why societies function in a coordinated manner or collapse under disjointed conflict [1,2]. Central to the theory is an argument for gene-level selection, and efforts to test its many predictions are befitting to population genetic data [3,4]. The mass adoption of genomic technologies into the field of social insect biology represents a timely transition towards new types of data that can build upon classic population genetics models to incorporate the broadest patterns of genetic and epigenetic variation [5,6,7]. This emerging field of sociogenomics has not yet been unequivocally aligned with inclusive fitness thinking [8,9], but there are opportunities to leverage the far-reaching theory against genomic and transcriptomic data sets.

In this study, we take a step in this direction by utilizing a termite transcriptome to examine how the nucleotide composition of expressed gene sets varies as a function of caste and population-level behaviour of the Eastern subterranean termite *Reticulitermes flavipes*. Termites are an all-eusocial clade (Termitoidea; ~3000 spp.) of cockroaches (Blattodea) that are distant relatives to any social Hymenoptera [10,11], and thus any member species presents a phylogenetically understudied opportunity to relate genomic profiles to social differences between individuals or populations [12,13]. The Eastern subterranean termite is a widely-studied species for which genomic resources are becoming more readily available, including a non-annotated draft genome [14]. This temperate North American species [15] is native to the eastern United States and south to contiguous parts of Mexico, but its affinity for human industry and habitation has contributed to its vicariant distribution elsewhere as a highly invasive pest [16,17].

**Figure 1:**
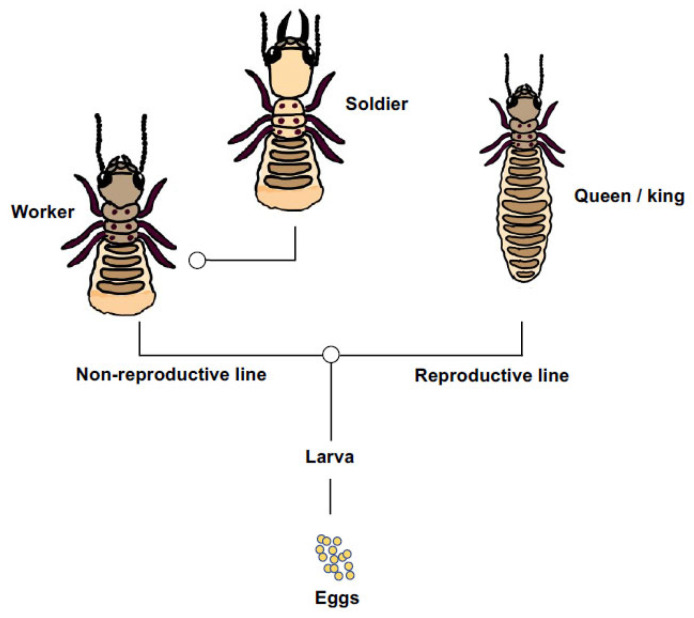
A simplified representation of caste developmental pathway in *Reticulitermes flavipes* [18,27]. Larvae differentiate into non-reproductive (worker and soldier) or reproductive (nymphs, primary queens and kings) castes. Workers can further differentiate into soldiers. These two points of caste differentiation or ‘switches’ sensu [28] are shown by open circles.

The basic social structure of the Eastern subterranean termite involves instars of both sexes that differentiate along one of two principal pathways, which are: the reproductive pathway from eggs and larva into wing-budded nymphs that can further differentiate into imagine queens and kings, and the non-reproductive pathway from eggs and larva into workers, which can further differentiate into soldiers (Figure 1). Because workers and soldiers have low or no direct fitness of their own and ultimately labour on behalf of reproducing relatives, they can rightly be considered reproductively altruistic [18,19]. As for most social insects, the process of caste differentiation is likely mediated by environmental cues that trigger physiological restrictions on development [20,21]. However, genetic differences too could influence an individual termite’s differentiation into one caste or another [22], and do so along either pathway. For *R. flavipes*, colonies are typically headed by a pair of outbred primary reproductives [23], but in invasive habitats the species tends toward a unicolonial structure with spatially diffuse nests, low aggression and rampant neotenic reproduction with potentially hundreds of egg-laying nymphs [24]. These changes to the social biology of invasive *Reticulitermes* may represent an exaggerated subsample of their native-range behaviour [25] but, regardless, unicoloniality makes invasive termites difficult to eradicate, with economic impact in North America scaling up to billions of dollars annually [26].

Similar to the process of caste differentiation, this social transition from colonial to unicolonial lifestyle may be mediated by environmental cues, but genetic bottlenecks and other sweeping changes to genetic diversity upon invasion may also play a role, especially if they dampen nestmate recognition cues that in turn lowers aggression [29]. Here, we exploit a series of cDNA libraries previously derived from whole-body tissue of three different castes as well as from behaviourally distinct colonial and unicolonial populations of the Eastern subterranean termite *Reticulitermes flavipes*. From caste- and population-specific libraries initially assembled by Wu et al. [30], we test for differences in expressed gene diversity between one reproductively selfish (wing-padded nymphs) and two reproductively altruistic (worker, soldier) castes. If caste differences are partly explained by genotype, then we expect caste-specific libraries to differ in the number, type or pattern of single nucleotide variants (SNVs). Likewise, if invasive transitions to unicoloniality in *R. flavipes* are associated with genetic bottlenecks or other systemic changes to nucleotide diversity, then we expect termites collected from invasive populations to harbour fewer SNVs or to otherwise show unique mutational profiles compared to their native counterparts. We test these two qualitative predictions by comparing the relative number and type of nucleotide substitutions against a consensus reference assembly.

## 2. Methods

### 2.1. Acquiring the RNA-Seq Data Set

**Figure 2:**
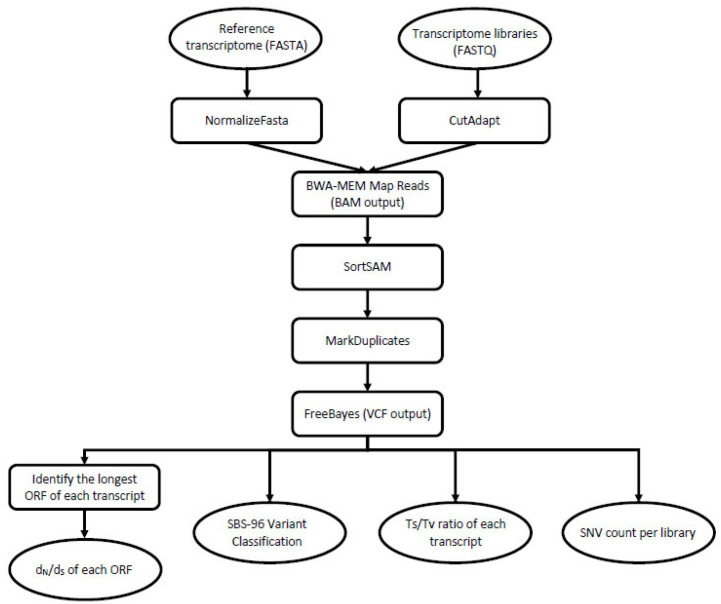
A graphical workflow depicting the major steps that we undertook in data processing. Oval shapes represent input/output, and the rectangular shapes represent the tools/processing steps. To begin, we accessed the nine NCBI-SRA library files (FASTQ format) and the corresponding reference assembly (FASTA format). From there, we used these data files in combination to generate the four principal analyses shown at the bottom of the flow chart.

The analyzed dataset consisted of nine RNA-Seq libraries, which were generated from three castes (nymph, soldier, worker). The samples include representatives of both sexes and were collected from each of three separate colonies across three geographic populations: one invasive (Toronto, ON, Canada) and two that are within the native range (near Raleigh, NC; near Boston, MA, USA). The details of sample collection, processing and sequencing are described in Wu et al. [30] and in Behl et al. [31]. For the purposes of this study, we accessed the National Center for Biotechnology Information Sequence Read Archive (NCBI-SRA) to download each of the nine termite transcriptome libraries (Table 1). Next, we transferred them onto the bioinformatics platform galaxy [32] for analysis. Each NCBI-SRA file consisted of paired sets of forward and reverse Illumina HiSeq 2000 sequence reads that correspond to a whole transcriptome of the cDNA template. Alongside each of these ~30 Gb fastq files, we used filezilla to transfer the corresponding reference assembly of 29,641 unique transcripts as a multi-fasta file into the galaxy environment. These and all other major steps in data analysis are shown in Figure 2.

### 2.2. Galaxy Workflow

Within galaxy, we configured a workflow to pre-process and map ~4.3 M sequence reads for automated variant calling. Our workflow adopted industry standard quality criteria [33]; it incorporated checking for Phred quality scores [34] and filtering out input files with incorrect or unreliable nucleotide base sequence calls that are sometimes generated due to the technical limitations of sequencing platforms. After this standardization step, we used the trinity method for the *de novo* assembly to map all suitable transcripts to a 14 K-gene reference transcriptome to visualize all aligned regions of reads, as in [35]. Following this, we produced variant call files (VCFs) that we screened for SNVs associated with population or caste. To reduce sequence-length bias, we employed normalizefasta tool to trim, to the same maximum length, all the reads aligned to the reference transcriptome. We further curated our dataset according to recommended practices [33] by implementing the cutadapt tool to locate and remove duplicate sequences from the fastq files.

The output files with reference transcriptome were then fed into the bwa-mem mapping tool, which makes use of the Burrow–Wheeler transform [36], and a block-sorting compression algorithm to generate alignments. Next, we removed duplicate sequences using markduplicates, then sorted the remaining sequences with the sortbam tool and compressed all aligned sequences into sets of binary bam files. By uploading bam files into the Integrative Genomics Viewer, we were able to visualize the reads mapped to the reference. To generate VCFs, we input the normalized reference and aligned-sequence bam files into freebayes [37], which implements Bayesian criteria to assess the likelihood of a variant call at each position in the transcriptome.

### 2.3. Analysis of Single Nucleotide Variation

To analyze generated VCFs, we first imported them into a local unix environment and performed analyses stepwise. We first used the splitvcfs tool available in the picard package of gatk [38] to isolate SNVs and exclude other variant types. Next, we implemented the countvariants tool built-in to gatk [38] to estimate the total number of SNVs for each population or caste-specific library. We then imported the compressed bgzip and indexed tabix VCFs into r. Next, within the rstudio environment, we used the vcfr [39], reshape2 [40] and ggplot2 [41] packages to filter out putative variants if the read depth was above a minimum threshold value of ‘10’, corresponding to our targeted minimum FDR value ≤ 0.1. Using r, we then tested if counts of SNVs differed between the three populations and castes.

To compare the broader mutational profile between caste- and population-specific libraries, we generated SBS-6 mutational catalogues that profiled the total count of all possible single base substitution mutation types referenced by the pyrimidine base (C > A, C > G, C > T, T > A, T > C and T > G) using a custom python script. We also developed SBS-96 mutational catalogues that profiled the count of all possible single base substitution mutation types and flanking 5′ and 3′ bases, for a total of 96 trinucleotide mutation types [42].

To quantify selection pressures using the *d_N_/d_S_* ratio of the entire transcriptome, we identified the longest open reading frame in each transcript bound by a start (AUG) and stop (UAA, UAG, UGA) codon. From this representative template, we estimated the proportion of synonymous (p_S_) and nonsynonymous (p_N_) substitutions by dividing the count of p_S_ and p_N_ for each transcript by the count of synonymous and nonsynonymous sites, respectively. Specifically, we estimated the number of synonymous (d_S_) and nonsynonymous (*d_N_*) substitutions per site using Equations (1) and (2) of Nei and Gojobori [43].

(1) $$d_{S}=-\frac{3}{4}ln\left(1-\frac{4p_{S}}{3}\right)$$

(2) $$d_{N}=-\frac{3}{4}ln\left(1-\frac{4p_{N}}{3}\right)$$

The *d_N_*/*d_S_* ratio was calculated by dividing *d_N_* by *d_S_* for each of the nine samples. The *d_N_*/*d_S_* ratio was also calculated from a subset of *n* = 570 genes that are known to strongly co-express with caste (minimum two-fold change and FDR < 0.05; [30]).

## 3. Results

**Figure 3:**
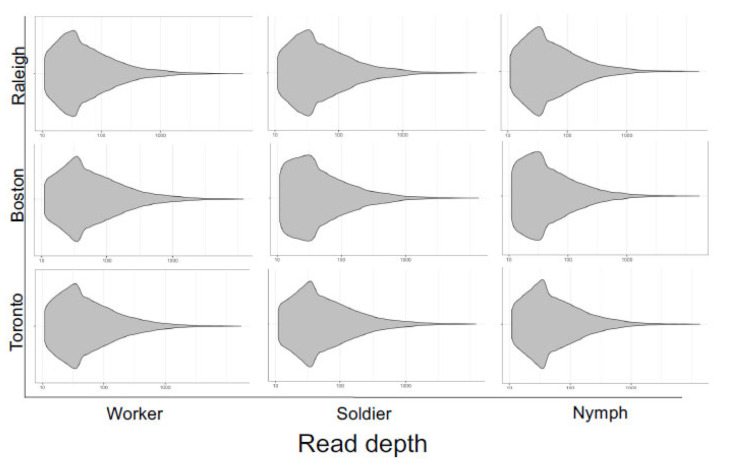
Read depth of high-quality (depth > 10) SNVs extracted from variant call files for three termite castes (worker, soldier, nymph) sampled from three populations (Raleigh, Boston, Toronto). The overall profiles of read depth coverage are similar between libraries, suggesting consistently high qualities.

We extracted SNVs from the whole-transcriptome libraries and compared the corresponding VCFs for each caste and population. The variant read depth coverage was comparable between all nine libraries (Figure 3) and revealed extensive SNV counts (~144 K SNVs per library; Table 1), especially in the 10–100 read depth range, indicating that the variants were inferred with low inter-sample bias and high technical accuracy [44].

**Figure 4:**
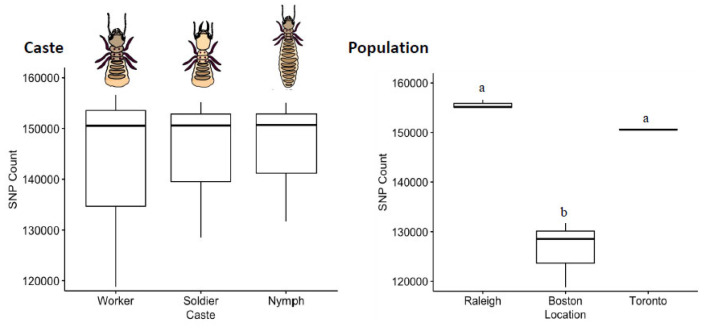
Counts of SNVs by caste and population. Termite castes are relatively uniform in SNV diversity. A pair-wise post-hoc analysis indicated that workers, soldiers and nymphs all harbour nearly identical levels of diversity in their transcribed sequences (Pairwise post-hoc Dunn Test, *p* > 0.4 in all cases). Among populations, however, termites collected from Raleigh were more variable than were those collected from Boston (Dunn tests; *p* = 0.0109), with the Toronto population being intermediate (Dunn test; *p* > 0.08 in both cases). Error bars are 95% CI. Different letters above boxes indicate a significant difference. Note that for the Toronto termite population, the three caste libraries used had a very similar SNV counts, and the estimate of variance is miniscule.

**Figure 5:**
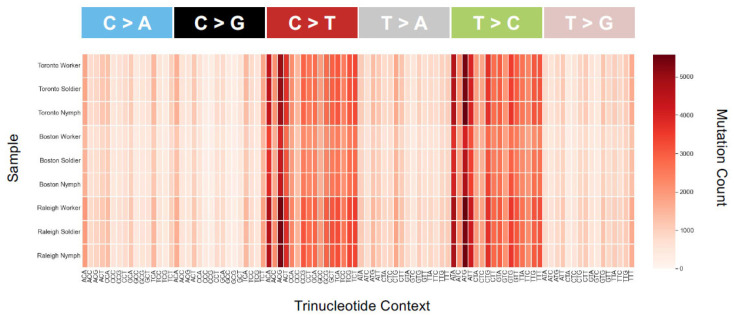
Heatmap of SBS-96 mutation counts for each of the nine samples. All 96 possible mutations are plotted (format available at https://cancer.sanger.ac.uk/cosmic/signatures/SBS, (accessed on 1 November 2021). Most plots show similar biases in mutation types and counts—for example, an excess of transitions across all samples. An SBS-96 plot with purine as a lead base is very similar qualitatively (not shown), consistent with similar mutational mechanisms across populations and castes. There are nonetheless slight differences in the actual numbers of SBSs between caste and population. Additionally, the scale for the Boston population has a smaller range than for Toronto and Raleigh, which is consistent with fewer variants found there (Figure 4B).

The distribution of SNVs did not differ by caste (Kruskal–Wallis Test, χ^2^ = 0.089, df = 2, *p* = 0.96; Figure 4). By comparison to a common reference transcriptome, worker, soldier and nymph libraries showed remarkably similar numbers of SNVs (~15 K), with the worker library having a slightly higher variance across the three sampled populations. The mean transition/transversion ratio was also nearly identical between castes (worker = 1.71, soldier = 1.70, nymph = 1.70; one-way ANOVA *F* = 0.68, *p* = 0.54), as were the overall SBS-96 mutational profiles (Figure 5).

The distribution of SNVs did differ by population (Kruskal–Wallis, χ_2_ = 7.2, df = 2, *p* = 0.027). By comparison to a common reference transcriptome, Raleigh and Toronto termites harbored significantly more expressed-gene diversity than termites sampled from Boston. Notably, the invasive unicolonial Toronto population was not the least diverse, and thus by comparative analysis does not show the obvious loss-of-diversity expected from a recent invasion bottleneck (Figure 4). The mean transition/transversion ratio was nearly identical among populations (Toronto = 1.69, Boston = 1.71, Raleigh = 1.70; one-way ANOVA *F* = 3.37, *p* = 0.104), though mutational profiles did reveal an excess of transitions (C > T and T > C) across all the nine libraries (Figure 5).

**Figure 6:**
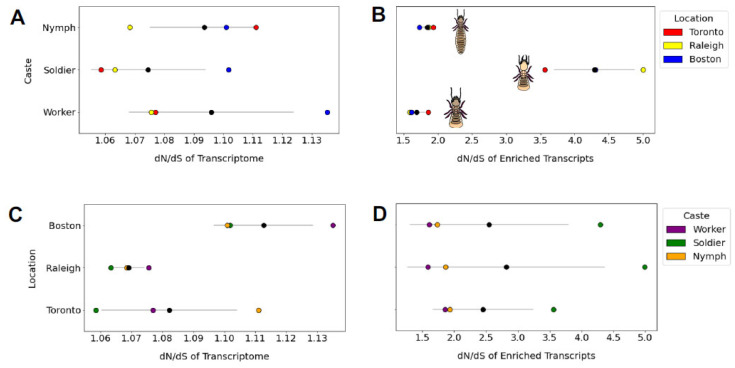
The *d_N_/d_S_* ratios of whole transcriptome and a subset of *n* = 230 caste-specific transcripts are grouped by caste or population. For each graph, mean and standard deviation are displayed as a black circle and grey line, respectively. We observed no transcriptome-wide differences in mean *d_N_/d_S_* between castes (shown in (**A**)). However, when caste-specific transcripts were tested separately (**B**), we found *d_N_/d_S_* of soldier-expressed genes to be significantly higher than *d_N_/d_S_* for genes uniquely expressed in nymphs (mean difference = 2.45, Tukey’s post hoc test *p* = 0.001) or workers (mean difference = 2.599, *p* = 0.01). We found no difference between populations in mean *d_N_/d_S_* at the whole-transcriptome (**C**) or caste-specific transcriptome (**D**) level.

The mean *d_N_/d_S_* ratio did not vary by population (one-way ANOVA *F* = 3.99, *p* = 0.079) or caste (one-way ANOVA *F* = 0.56, *p* = 0.601; Figure 6). However, when we performed this same analysis on a 230-gene subset of caste-associated genes identified by Wu et al. (2018), the *d_N_/d_S_* ratio did differ by caste (one-way ANOVA *F* = 35.004, *p* = 0.0004) but not by population (one-way ANOVA *F* = 0.047, *p* = 0.95). Specifically, genes associated in their expression with the defensively specialized and sterile soldier caste appear to evolve at a higher rate (*d_N_/d_S_* = 4.3) than genes associated with the nymph (*d_N_/d_S_* = 1.8) or worker (*d_N_/d_S_* = 1.7) castes (Figure 6).

## 4. Discussion

In this study, we used RNA-Seq analysis to reveal genetic events associated with caste and invasiveness from comparably sampled North American populations of the Eastern subterranean termite. Our whole transcriptome-wide screen, spanning 29,641 transcripts that represent 13,755 genes, showed a remarkably consistent SNV count for each caste library, but the nature of these variants suggest that some confer a caste-specific advantage. A codon-based analysis revealed a relatively high *d_N_/d_S_* ratio (~4.3) in genes associated with the reproductively altruistic soldier caste, regardless of the population from which the individuals were sampled. Given that soldiers are sterile and thus have no direct fitness, any evidence for selection on genes functionally associated with this caste is de facto evidence for indirect selection, of the type mediated through kin selection. Additionally, we found differences in SNV diversity across three termite populations but not in the manner expected if the invasive population was recently bottlenecked. The Toronto population was not the least diverse; it harboured a similar or higher number of SNVs as one of the native range populations without revealing an obvious mark of a bottleneck or any other substitution patterns associated with the invaded habitat. We suggest that a 90-year timeframe, since the Eastern subterranean termite invasion of the city of Toronto, may be sufficient to obscure any obvious signature of a past bottleneck. By extension, we suggest that the unicolonial behaviour of invasive termites observed in the city is no longer strictly associated with the loss of genetic diversity.

### 4.1. Test for Caste Differences

Despite a consensus that genetic effects on social insect caste ought to be rare, we nonetheless find occasional and sometimes complex exceptions to this rule [45,46], including from termites [47,48] and specifically from *Reticulitermes* [25,49,50]. There are a growing number of examples of weak or strong genetic effects on caste and subcaste differentiation. This is especially evident in social insect species that mate more than once (i.e., polyandry), have loci that constrain development into one caste or another, hybridize between reproductively isolated lineages, or recurrently use some form of thelytokous pathenogenesis to clone the queen [28]. Genotype-caste associations can therefore evolve in strikingly different and complex ways.

For our focal species, environmentally mediated gene expression is an important and well-established factor in caste differentiation [30,51,52], yet no test has prior examined how variants of expressed genes might correlate with caste. We found that our selected measure of nucleotide diversity (i.e., the number of SNVs) does not covary with caste, at least not in the three castes sampled from three populations. Instead, variation levels within and between castes are similar. Therefore, no transcriptome-wide association between caste and SNVs at either of the developmental switches was detected in *R. flavipes* caste development. That is, there is no widespread difference in RNA diversity between castes representative of the reproductive (nymph) and non-reproductive lines (workers, soldiers), or between the workers and soldiers themselves. The *d_N_/d_S_* analysis did, however, reveal an intriguing difference between caste-associated genes. Specifically, a subset of genes known to associate in their expression with the soldier caste differed in *d_N_/d_S_* ratio from genes uniquely expressed in the nymph and worker caste. This caste-biased pattern in nucleotide substitution suggests that selection acts differently on genes that are functionally associated with different castes. Our analysis does not include primary reproductive—‘kings’ and ‘queens’. First, these castes can be difficult to locate and extract from diffuse underground nests but, also, they are effectively absent in northern and invasive populations such as Toronto. In our study, we use late-instar nymphs to represent the (neotenic) reproductive caste.

Prior studies demonstrate that soldier-biased genes are taxonomically restricted [31] and enriched for biologically novel processes [30]. These patterns further indicate that genes associated with the soldier caste in *Reticulitermes* evolve under grossly different selection pressures than genes associated with other castes. The Nei and Gojobori estimate of selection was developed well before population genomic data. Our use of this estimate here may therefore differ from its initially intended use and violate some of its assumptions regarding the nature of mutations [53], which in our case are SNVs. Its simple formulae nonetheless remain in widespread use and provides an intuitive measure of selection. The precise signature of caste-mediated selection is not yet well characterised for termites [54] or any social insect [55], but our RNA-Seq analysis clearly reveals evidence for differential selection of soldier-associated genes. Why soldier-associated genes have a two-fold higher estimate of *d_N_/d_S_* than worker- or nymph-associated genes in our dataset remain unknown but appears to be unrelated to habitat and therefore unrelated to invasion. Nevertheless, soldiers of *Reticulitermes* have no reproductive potential of their own—they are sterile—and thus gain fitness only indirectly through assisted production of non-descendent kin (i.e., siblings, half-siblings, etc.). The novel evidence for selection on soldier-expressed genes in this study is presumably indirect in nature and commonly referred to as kin selection.

### 4.2. Test for Population Differences

*Reticulitermes* are typically colonial and thus live in simple or extended families [25], yet for some populations nestmate recognition appears weak or absent, notably in populations invasive to France [29,56] or other parts of the world [17]. In the city of Toronto, and other Canadian municipalities where *R. flavipes* has invaded [57], the unicolonial populations are characterized, as in France [56], by low aggression and apparent intermixing of colonies that lack clear kin boundaries [58,59]. Moreover, unicolonial termite populations retain a high capacity for secondary neotenic reproduction via brachypterous (wing-budded) nymphs that can form independently reproductive sub-colonies [23]. These invasive qualities from a social insect [60,61] likely contribute to the success of *R. flavipes* in urban environments, rendering them more difficult to eradicate.

Early research suggests that the introduction of *R. flavipes* to the city of Toronto was a single event along the city’s industrial foreshore between the years 1935 and 1938 [62]. How many reproductives were introduced to ‘the shores of Toronto Bay’ is not known, but whatever genetic diversity is currently present in this large metropolitan population of termites may have arisen from a bottleneck that occurred over the last ~90 years. Previous genetic analysis of microsatellite DNA showed that the termites of Toronto are genetically distinct from other subterranean termite populations in the province, e.g., [63] and have presumably spread across the city’s metropolitan area (~630 sq. km) via nest budding and human-mediated dispersal [64]. Despite the invasiveness of the urban-dwelling termites of Toronto, our analysis reveals as many or more polymorphic transcripts than southernly forest-dwelling populations in the species’ native range. Time, large effective populations sizes and the possibility of repeated invasions from the United States [64] or secondary invasions via Europe [17] may therefore have restored any initial loss of diversity upon invasion.

In our study, we have used a de novo consensus assembly as a generic reference for all comparisons. At present, no annotated model genome that might have served as an out-of-study neutral comparator is available for *R. flavipes*. Because our reference sequence does not correspond to any single individual, but rather is a consensus sequence generated from multiple library reads, we do not attribute importance to any specific functional difference. We do, however, identify broad patterns of diversity that qualitatively distinguish libraries and serve to test our predictions for one population or caste to have fewer (or more) silent or non-silent SNVs than another. Collectively, our findings suggest that unicolonialism in the one invasive population studied here is actually not linked to any single transcriptome-wide pattern of diversity, as may be the case elsewhere for *Reticulitermes* [29,65] or for other social insects [66,67,68]. Instead, unicoloniality in Toronto termites may depend on other aspects of their biology that we have not measured here. Potentially, these may include flexible social organization [60], cold tolerance [69], diversity in cues involved in nestmate recognition [70] and ecological conditions that may have promoted unicoloniality [71]. For example, short summers at the northern latitudes of Ontario may select for local reliance on relatively rapid neotenic reproduction within natal nests, instead of the production of dispersing alates and nest founding [72]. Further, the absence of primary sexuals in unicolonies may in turn dampen the normal pheromone-induced incentive for nestmate recognition and inter-colony defence [73], resulting in relatively ‘open’ societies [74] that consist of interconnected nests with mixed kin groups. Our work thus suggests an important role for both environmental (climatic constraints) and social (pheromonal) factors that are implicated in maintaining unicolony formation in northern invasive subterranean termites.

## Figures and Tables

**Table 1:** Summary statistics for *Reticulitermes flavipes* SNV count by population and caste.

	SRA File Accession	SNV Count Mean ± SD
Caste		
Nymph	SAMN06579168 SAMN06579169 SAMN06579170	145,824 ± 12,414
Soldier	SAMN06579171 SAMN06579172 SAMN06579173	144,752 ± 14,243
Worker	SAMN06579174 SAMN06579175 SAMN06579176	141,981 ± 20,306
Population		
Raleigh	SAMN06579175 SAMN06579172 SAMN06579169	155,606 ± 870
Boston	SAMN06579174 SAMN06579171 SAMN06579168	126,342 ± 6726
Toronto	SAMN06579176 SAMN06579173 SAMN06579170	150,608 ± 86.4

## Data Availability

Data used in this study are publicly available through the National Center for Biotechnology Information Sequence Read Archive (NCBI-SRA) under the accession numbers presented in Table 1.
